# PFKFB3 gene deletion in endothelial cells inhibits intraplaque angiogenesis and lesion formation in a murine model of venous bypass grafting

**DOI:** 10.1007/s10456-021-09816-3

**Published:** 2021-08-25

**Authors:** Paola Perrotta, Margreet R. de Vries, Bart Peeters, Pieter-Jan Guns, Guido R. Y. De Meyer, Paul H. A. Quax, Wim Martinet

**Affiliations:** 1 Laboratory of Physiopharmacology, University of Antwerp, Universiteitsplein 1, B-2610 Antwerp, Belgium; 2 Department of Surgery, Leiden University Medical Center, Albinusdreef 2, 2333 ZA Leiden, The Netherlands; 3 Laboratory Medicine, Antwerp University Hospital, Drie Eikenstraat 655, B-2650 Edegem, Belgium

**Keywords:** Vein graft, Atherosclerosis, Intraplaque neovascularization, Glycolysis, PFKFB3

## Abstract

Vein grafting is a frequently used surgical intervention for cardiac revascularization. However, vein grafts display regions with intraplaque (IP) angiogenesis, which promotes atherogenesis and formation of unstable plaques. Graft neovessels are mainly composed of endothelial cells (ECs) that largely depend on glycolysis for migration and proliferation. In the present study, we aimed to investigate whether loss of the glycolytic flux enzyme phosphofructokinase-2/fructose-2,6-bisphosphatase 3 (PFKFB3) in ECs inhibits IP angiogenesis and as such prevents unstable plaque formation. To this end, apolipoprotein E deficient (ApoE^−/−^) mice were backcrossed to a previously generated PFKFB3^fl/fl^ Cdh5^iCre^ mouse strain. Animals were injected with either corn oil (ApoE^−/−^PFKFB3^fl/fl^) or tamoxifen (ApoE^−/−^PFKFB3^ECKO^), and were fed a western-type diet for 4 weeks prior to vein grafting. Hereafter, mice received a western diet for an additional 28 days and were then sacrificed for graft assessment. Size and thickness of vein graft lesions decreased by 35 and 32%, respectively, in ApoE^−/−^PFKFB3^ECKO^ mice compared to controls, while stenosis diminished by 23%. Moreover, vein graft lesions in ApoE^−/−^PFKFB3^ECKO^ mice showed a significant reduction in macrophage infiltration (29%), number of neovessels (62%), and hemorrhages (86%). EC-specific PFKFB3 deletion did not show obvious adverse effects or changes in general metabolism. Interestingly, RT-PCR showed an increased M2 macrophage signature in vein grafts from ApoE^−/−^PFKFB3^ECKO^ mice. Altogether, EC-specific PFKFB3 gene deletion leads to a significant reduction in lesion size, IP angiogenesis, and hemorrhagic complications in vein grafts. This study demonstrates that inhibition of endothelial glycolysis is a promising therapeutic strategy to slow down plaque progression.

**Supplementary Information:**

The online version contains supplementary material available at 10.1007/s10456-021-09816-3.

## Introduction

Atherosclerosis is a chronic inflammatory disease of the arterial wall and it is one of the most important causes of cardiovascular disease, including severe conditions such as coronary artery disease, myocardial infarction, heart failure, and stroke. Vein bypass grafting is a surgical procedure that uses large saphenous veins to bypass occluded atherosclerotic arteries, thereby allowing revascularization of an ischemic region of the heart or limbs [1]. Unfortunately, at least 40% of patients suffer from bypass failure within eight years after the procedure due to negative vascular remodeling and intimal hyperplasia [1–4]. Furthermore, vein grafts often present accelerated atherosclerosis with formation of unstable plaques and increased risk of rupture [5–7].

New small vessels can form inside vein grafts to fulfill an increased demand for oxygen and nourishment of the vessel wall. This event, which is further promoted by inflammatory conditions, leads to intraplaque (IP) angiogenesis and contributes to plaque instability in the vein graft [8, 9]. Indeed, apolipoprotein E deficient (ApoE^−/−^) mice undergoing a vein graft interposition of the carotid artery develop unstable plaques with extensive IP neovessels that are often dysfunctional or immature and contribute to lesion destabilization by enhancing leukocyte recruitment and accumulation of cholesterol and platelets [10, 11].

Angiogenesis is an energy-intensive process that requires extensive metabolic functioning of endothelial cells (ECs) to support sprouting, migration, and proliferation [12]. Recent studies have shown that ECs in neovessels generate more than 85% of their ATP by glycolysis [13, 14]. One of the rate-limiting checkpoints of glycolytic flux is the conversion of fructose-6-phosphate to fructose-1,6-bisphosphate by 6-phosphofructo-1-kinase. Phosphofructokinase-2/fructose-2,6-bisphosphatase (PFKFB) enzymes synthesize fructose-2,6-bisphosphate, an allosteric activator of 6-phosphofructo-1-kinase and the most potent stimulator of glycolysis. Of all PFKFB iso-enzymes, PFKFB3 appears the major producer of intracellular fructose-2,6-bisphosphate in ECs. PFKFB3 is upregulated in ECs under inflammatory conditions and its pharmacological inhibition or gene silencing reduces pathological angiogenesis in response to injury and inflammation [15–17]. Previous findings have shown that inhibition of PFKFB3 leads to reduced EC migration and proliferation in vitro. Additionally, sprout number and length of EC spheroids significantly decrease after knocking out PFKFB3 [18].

We have recently reported that the partial glycolysis inhibitor 3PO [3-(3-pyridinyl)-1-(4-pyridinyl)-2-propen-1-one] reduces IP angiogenesis and plaque formation [19]. However, the specific role of endothelial PFKFB3 in the context of IP neovascularization and lesion progression remains to be investigated. Therefore, in the present study we used a vein graft procedure in EC-specific conditional PFKFB3 knockout mice on an ApoE^−/−^ background to test whether endothelial PFKFB3 is an important driver of IP angiogenesis and atherosclerotic lesion progression.

## Materials and methods

### Animals

EC-specific conditional PFKFB3 knockout mice (PFKFB3^fl/fl^) were generated by crossbreeding PFKFB3^fl/fl^ mice with VE-cadherin (PAC)-Cre^ERT2^ mice (Cdh5^iCre^) [18]. Resulting mice were crossbred with ApoE^−/−^ mice to generate an ApoE^−/−^PFKFB3^fl/fl^Cdh5^iCre^ strain. All mice were on a C57BL/6 N background. ApoE^−/−^PFKFB3^fl/fl^Cdh5^iCre^ mice (male, 6 weeks old) were injected with tamoxifen (0.1 g/kg body weight) for 5 consecutive days to induce PFKFB3 deletion in ECs, termed ApoE^−/−^PFKFB3^ECKO^. ApoE^−/−^PFKFB3^fl/fl^Cdh5^iCre^ control mice, further referred to as ApoE^−/−^PFKFB3^fl/fl^ mice, were injected with corn oil using the same protocol. All animal procedures were conducted according to the guidelines from Directive 2010/63/EU of the European Parliament on the protection of animals used for scientific purposes. Experiments were approved by the ethics committee of the University of Antwerp (reference number 2017-96).

### Vein graft surgery

**Fig. 1:**
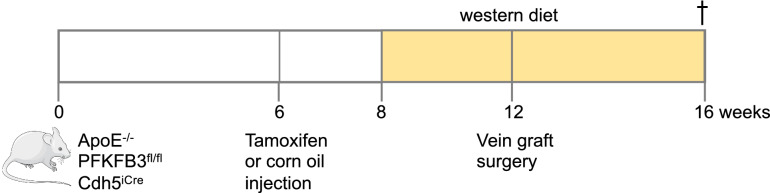
Schematic overview of the experimental design. ApoE^−/−^PFKFB3^fl/fl^Cdh5^iCre^ mice (6 weeks old) were injected with tamoxifen (0.1 g/kg body weight) for 5 consecutive days to induce PFKFB3 deletion in ECs. Control mice were injected with corn oil using the same protocol. After 2 weeks, mice were fed a western-type diet. Four weeks later, vein graft surgeries were performed. Mice were sacrificed 4 weeks after surgery

ApoE^−/−^PFKFB3^ECKO^ and ApoE^−/−^PFKFB3^fl/fl^ mice were fed a western-type diet (Altromin, C1000 diet supplemented with 20% milkfat and 0.15% cholesterol, #100,171) for 4 weeks (Fig. 1). Next, vein graft surgery was performed as described [5, 7, 9]. Briefly, thoracal caval veins from donor ApoE^−/−^PFKFB3^ECKO^ or ApoE^−/−^PFKFB3^fl/fl^ mice were harvested. In the first group, ApoE^−/−^PFKFB3^fl/fl^ recipient mice received the caval veins from ApoE^−/−^PFKFB3^fl/fl^ donor mice; in the second group, ApoE^−/−^PFKFB3^ECKO^ mice received the caval veins from ApoE^−/−^PFKFB3^ECKO^ mice. For each experiment, the right carotid artery of recipient mice was dissected and cut in the middle. On both the proximal and distal artery end, a nylon cuff was sleeved and fixated with hemostatic clamps. The artery was everted around the cuffs and ligated with 8.0 sutures. Next, the caval veins were positioned over both cuffs, and ligated. Before surgery, mice were anesthetized with midazolam (5 mg/kg body weight, i.p., Roche), medetomidine (0.5 mg/kg body weight, i.p., Orion) and Fentanyl (0.05 mg/kg body weight, i.p., Janssen). After the procedure, mice were antagonized with atipamezole (2.5 mg/kg body weight, i.p., Orion) and fluminazenil (0.5 mg/kg body weight, i.p., Fresenius Kabi). Buprenorphine (0.1 mg/kg body weight, i.p., MSD Animal Health) was given after surgery to relieve pain. Animals were sacrificed under the aforementioned anesthesia 28 days after the graft procedure, followed by 2 min of in vivo perfusion-fixation.

### Histology

After euthanasia, vein graft segments were collected, fixed in 4% paraformaldehyde (PFA) for 24 h, dehydrated overnight in 60% isopropanol, and subsequently embedded in paraffin. Cross sections of vein graft segments were stained with hematoxylin and eosin to evaluate lumen and lesion area, plaque thickness, and percentage of vein stenosis. Neovessels were detected inside vein graft lesions via standard immunohistochemistry using anti-CD31 antibody (endothelial cells; ab124432, Abcam). Anti-TER-119 (550,565, BD Biosciences) was used to determine plaque hemorrhages and anti-α-smooth muscle actin (α-SMA) (A2547, Sigma-Aldrich) was used to determine vascular smooth muscle cell (VSMC) coverage of neovessels. Anti-MAC3 (550,292, Pharmingen), a Masson’s Trichrome stain and anti-vascular cell adhesion molecule-1 (VCAM-1) (ab134047, Abcam) were used to stain macrophages, collagen, and VCAM-1 positive ECs, respectively.

### Metabolic parameters

To determine whether ApoE^−/−^PFKFB3^ECKO^ mice exhibit an alteration in glucose metabolism and to characterize the metabolic phenotype, a glucose tolerance test (GTT) and insulin tolerance test (ITT) were done. To perform GTT, mice were fasted for 16 h, injected with a single dose of glucose (1 g/kg body weight, i.p.) and then glucose levels in peripheral blood (from tail) were determined after fixed time intervals (0–30–60–120 min) using a hand-held glucometer (OneTouch Ultra, range 20–600 mg/dL; Lifescan). For ITT, a single insulin dose was injected (Novorapid, 1 U/kg body weight, i.p.) in mice and blood glucose levels were monitored as in GTT. Liver enzymes, total cholesterol, and triglycerides were analyzed with an automated Vista 1500 System (Siemens Healthcare Diagnostics). Insulin and β-hydroxybutyrate in plasma samples were determined with a mouse insulin ELISA kit (80-INSMS-E01, ALPCO) and β-hydroxybutyrate assay kit (ab83390, Abcam), respectively.

### Aortic sprouting

An aortic ring assay was performed as previously described [20, 21]. In brief, murine thoracic aortas were dissected, cleaned under sterile conditions, transferred to 10 cm culture dishes, and cut into 0.5 mm thick rings with a sterile scalpel. After overnight starvation in serum-free Opti-MEM at 37 °C, ring segments were transferred into wells of a 96-well plate coated with 50 µL of a freshly prepared collagen type I solution (1 mg/mL). The aortic rings remained in Opti-MEM (supplemented with 2.5% fetal bovine serum and antibiotics) in the presence or absence of vascular endothelial growth factor (40 ng/mL, R&D Systems). Medium was replaced every 2 days. On day 6, rings were fixed with 4% paraformaldehyde and stained with von Willebrand factor antibody (PC054, Binding Site) that was added overnight prior to fluorescence microscopy imaging. The number of sprouts was counted for each ring and sprout numbers per ring were averaged for each group and graphed.

### Mouse lung EC isolation

In order to check the efficiency of PFKFB3 deletion after tamoxifen injection, primary mouse lung ECs were isolated as previously described [22, 23]. Briefly, 4 lungs were harvested, finely minced with scissors, and digested with 1.5 mg/ml collagenase Type I (Sigma-Aldrich #C0130) at 37 °C for 45 min (under gentle agitation). The digested cell suspension was filtered on a 70 µM sterile cell strainer, and spun at 400 g for 10 min. The pellet was resuspended in 2 ml of 0.1% bovine serum albumin and 50 µL magnetic dynabeads (ThermoFisher #11,035) precoated overnight with anti-mouse CD31 (BD Pharmingen #553,370) for EC-positive selection. After 20 min at room temperature under slow rotation, the bead-bound cells were recovered with a magnetic separator and washed five times with DMEM containing 10% fetal bovine serum. Cells were finally resuspended in 10 mL of complete DMEM medium (DMEM containing 20% fetal bovine serum, endothelial cell growth supplement, and antibiotics), seeded onto gelatin-precoated 10 cm plates, and grown for a few days to obtain enough protein material for western blotting.

### Western blot analyses

Cells were lysed in an appropriate volume of Laemmli sample buffer (Bio-Rad) containing β-mercaptoethanol (Sigma-Aldrich) and boiled for 5 min. Protein samples were then loaded onto pre-casted Bolt 4–12% Tris-Bis gels (Invitrogen) and after electrophoresis transferred to Immobilon-FL PVDF membranes (Millipore) according to standard procedures. Membranes were blocked for 1 h with Odyssey blocking buffer (LI-COR Biosciences) diluted 1:5 with PBS. After blocking, membranes were probed overnight at 4 °C with primary antibodies diluted in Odyssey blocking buffer, followed by 1 h incubation with IRDye-labeled secondary antibodies at room temperature. Antibody detection was achieved using an Odyssey SA infrared imaging system (LI-COR Biosciences). The intensity of the protein bands was quantified using Image Studio software. The following primary antibodies were used: anti-β-actin (ab8226, Abcam) and anti-PFKFB3 (ab181861, Abcam). IRDye-labeled secondary antibodies (goat anti-mouse IgG, 926-68070, and goat anti-rabbit IgG, 926-32211) were purchased from LI-COR Biosciences.

### Real-time RT-PCR

Total RNA was isolated from formalin-fixed, paraffin-embedded tissue sections using an RNeasy FFPE kit (Qiagen) according to the manufacturer’s instructions. Reverse transcription was performed with a Sensifast™ cDNA Synthesis Kit (Bioline). Thereafter, Taqman gene expression assays (Applied Biosystems) for CD38 (assay ID: Mm01220906_m1), Gpr18 (assay ID: Mm01224541_m1), Egr2 (assay ID: Mm00456650_m1), Arg1 (assay ID: Mm00475988_m1), CXCR4 (assay ID: Mm01292123_m1), VEGFA (assay ID: Mm00437306-m1), VCAM-1 (assay ID: Mm01320970_m1), and ICAM-1 (assay ID: Mm00516023_m1) were performed in duplicate on a QuantStudio 3 real-time PCR system (Applied Biosystems). The parameters for PCR amplification were 95 °C for 10 min followed by 40 cycles of 95 °C for 15 s and 60 °C for 1 min. Relative expression of mRNA was calculated using the comparative threshold cycle method. All data were normalized for quantity of cDNA input by performing measurements on the endogenous reference gene β-actin (assay ID: Mm00607939_s1).

### Statistics

All data are expressed as mean ± SEM. Statistical analyses were performed using GraphPad Prism (version 9) and SPSS (version 25) software. Statistical tests are specified in the figure legends. Differences were considered significant at *P* < 0.05.

## Results

### ECs of ApoE^−/−^PFKFB3^ECKO^ mice are PFKFB3 deficient

**Fig. 2:**
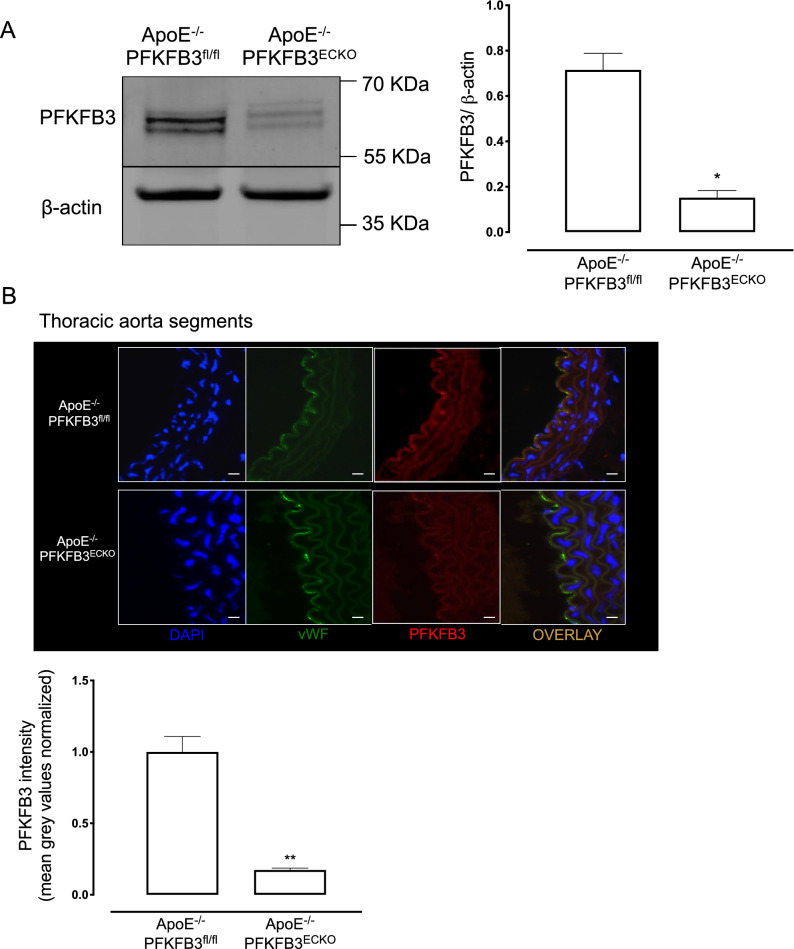
Validation of PFKFB3 gene deletion in endothelial cells. **A** Representative western blot showing protein levels of PFKFB3 and β-actin in primary lung EC. Bars represent relative protein quantification of PFKFB3 normalized to the reference protein β-actin. *P < 0.05 versus ApoE^−/−^PFKFB3^fl/fl^ (Independent samples *t* test; *n* = 3). **B** Representative thoracic aorta segments of ApoE^−/−^PFKFB3^fl/fl^ and ApoE^−/−^PFKFB3^ECKO^ stained with antibodies against PFKFB3 or von Willebrand Factor (vWF; endothelial cell marker). Nuclei were stained with DAPI. Scale bar = 100 μm. PFKFB3 signal in vWF positive cells was quantified. **P < 0.01 versus ApoE^−/−^PFKFB3^fl/fl^ (Independent samples *t* test; *n* = 3)

ApoE^−/−^PFKFB3^fl/fl^Cdh5^iCre^ mice were injected with corn oil or tamoxifen to generate ApoE^−/−^PFKFB3^fl/fl^ control mice and ApoE^−/−^PFKFB3^ECKO^ mice (with PFKFB3 specifically deleted in ECs), respectively (Fig. 1). To assess the efficiency of PFKFB3 deletion after tamoxifen injection, lung ECs from ApoE^−/−^PFKFB3^fl/fl^ and ApoE^−/−^PFKFB3^ECKO^ mice were isolated and examined by western blotting. PFKFB3 protein levels were reduced by more than 80% in ApoE^−/−^PFKFB3^ECKO^ mice (Fig. 2A). Similar findings were observed after co-staining of thoracic aorta segments with anti-PFKFB3 and anti-von Willebrand factor antibodies (Fig. 2B).

### ECs of ApoE^−/−^PFKFB3^ECKO^ mice show impaired sprouting in an ex vivo mouse aortic ring assay

Previous findings have shown that inhibition of PFKFB3 leads to a reduction of EC migration and proliferation in vitro [18]. In line with these results, we found that vascular endothelial growth factor-induced sprouting in aortic rings of ApoE^−/−^PFKFB3^ECKO^ mice was 65% less as compared to ApoE^−/−^PFKFB3^fl/fl^ control mice (Supplemental Figure S1). This observation indicates a direct effect of endothelial PFKFB3 on angiogenesis.

### PFKFB3 deficiency in ECs does not cause metabolic changes in adult mice

To evaluate whether EC-specific PFKFB3 gene deletion affects general metabolism, we analyzed plasma samples of ApoE^−/−^PFKFB3^fl/fl^ and ApoE^−/−^PFKFB3^ECKO^ mice after 4 weeks of western-type diet. No differences were observed in liver enzymes (γ-glutamyltransferase, alanine transaminase, alkaline phosphatase) and insulin (Table 1), indicating that PFKFB3 deletion in ECs has no obvious systemic side effects. Moreover, there were no differences in glucose and insulin tolerance tests after 12 weeks of western-type diet (Supplemental Figure S2), indicating that glucose absorption and insulin receptor sensitivity is normal in both strains. Also, body weight, cholesterol levels, and plasma triglycerides were not statistically different between both groups of mice (Table 1). In addition, levels of the ketone-body β-hydroxybutyrate were not changed in ApoE^-/-^PFKFB3^ECKO^ mice as compared to ApoE^−/−^PFKFB3^fl/fl^ mice (Table 1). These observations suggest that endothelial PFKFB3 deletion does not induce a metabolic switch from glucose to fatty acid–derived ketones and does not cause major side effects in ApoE^−/−^ mice.

**Table. 1:** Metabolic parameters of ApoE^−/−^PFKFB3^fl/fl^ and ApoE^−/−^PFKFB3^ECKO^ mice

Metabolic parameters	ApoE^−/−^PFKFB3^fl/fl^	ApoE^−/−^PFKFB3^ECKO^
Liver enzymes		
γ-Glutamyltransferase (U/L)	5.5 ± 0.6	5 ± 0.4
Alanine transaminase (U/L)	39 ± 11	34 ± 6
Alkaline phosphatase (U/L)	132 ± 13	154 ± 13
Fasting blood glucose (mg/dL)	116 ± 4	100 ± 8
Non-fasting blood glucose (mg/dL)	152 ± 9	138 ± 10
Insulin (ng/ml)	0.2 ± 0.1	0.2 ± 0.1
Total cholesterol (mg/dL)	306 ± 2	317 ± 1
Body weight (g)	20 ± 0.4	19 ± 0.3
Triglycerides (mg/dL)	79 ± 23	57 ± 6
β-hydroxybutyrate (µM)	1.9 ± 0.2	2.0 ± 0.1

Data between ApoE^−/−^PFKFB3^ECKO^ and ApoE^−/−^PFKFB3^fl/fl^ mice are not significantly different. Independent Sample *t* test, *n* = 8 (ApoE^−/−^PFKFB3^fl/fl^) or *n* = 11 (ApoE^−/−^PFKFB3^ECKO^)

### PFKFB3 deficiency in ECs inhibits neovascularization in vein graft lesions

**Fig. 3:**
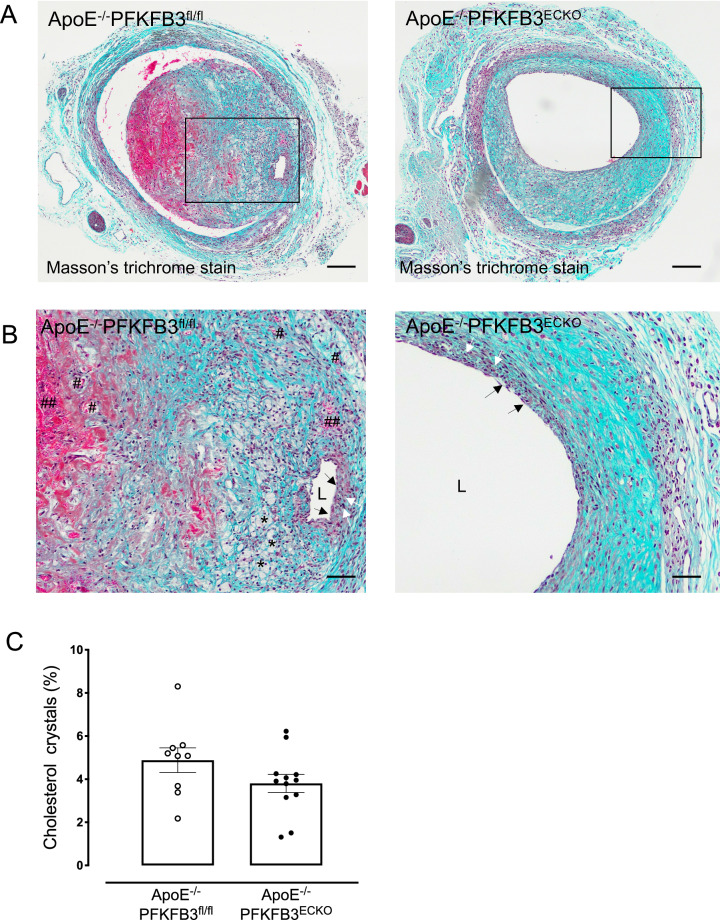
PFKFB3 deletion in endothelial cells evokes a more stable vein graft lesion phenotype. **A** Masson’s Trichrome staining of representative vein grafts. This staining was used for the evaluation of lesion complexity. Nuclei are stained dark blue, erythrocytes are stained bright-red, and collagen is stained blue. The staining allows easy visualization of intraplaque hemorrhages (red areas) as well as necrotic core and cholesterol crystals (white acellular regions). Scale bar = 200 μm. **B** Boxed area of panel A showing an almost intact endothelium exposed to the lumen (L) (black arrows), a fibrous cap with numerous vascular smooth muscle cells (white arrows), small foam cells (asterisks), an area with extravasated erythrocytes (##), and neovascularization (#). Scale bar = 100 μm. **C** Quantification of foam cells in vein graft lesions of ApoE^−/−^PFKFB3^fl/fl^ and ApoE^−/−^PFKFB3^ECKO^ mice. Independent samples *t* test; *n* = 9 (ApoE^−/−^PFKFB3^fl/fl^) or *n* = 12 (ApoE^−/−^PFKFB3^ECKO^ )

Both ApoE^−/−^PFKFB3^fl/fl^ and ApoE^−/−^PFKFB3^ECKO^ mice displayed an intact endothelium 28 days after vein graft surgery (black arrows Fig. 3A, B). Circular-oriented VSMCs were seen in ApoE^−/−^PFKFB3^fl/fl^ and ApoE^−/−^PFKFB3^ECKO^ vein graft lesions, close to the lumen, suggesting a cap-like organization (white arrows, Fig. 3A, B). Foam cells, a small necrotic core, and cholesterol crystals were found particularly in vein grafts of ApoE^−/−^PFKFB3^fl/fl^ mice near the luminal side (asterisks, Fig. 3A, B). Furthermore, neovessels were found through the vein graft wall, predominantly in ApoE^−/−^PFKFB3^fl/fl^ mice. The newly formed vessels were often leaky as extravasated erythrocytes were found near and outside these microvessels (Fig. 3A, B).

**Fig. 4:**
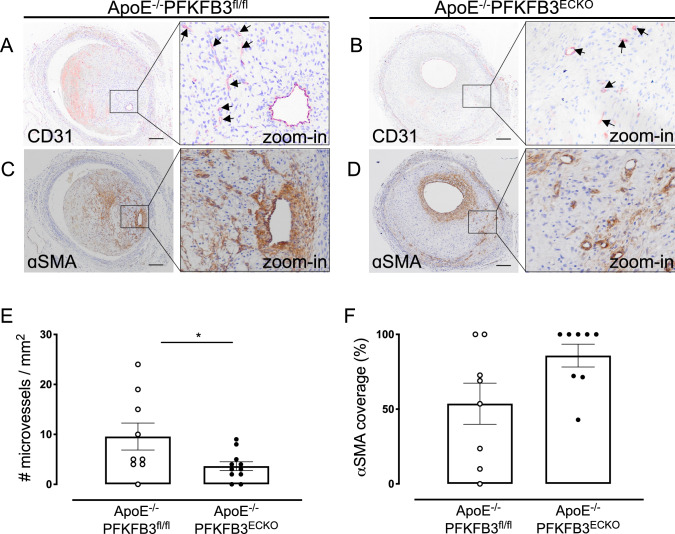
PFKFB3 deficiency in endothelial cells inhibits intraplaque neovascularization in vein graft lesions but does not alter α-SMA coverage of microvessels. **A** and **B** Representative vein graft lesions stained with CD31 antibody. Endothelial cells are detected as CD31 positivity (dark violet) around the vessel lumen. Microvessels are marked by arrows. Scale bar = 200 μm. **C** and **D** Representative vein graft lesions stained with anti-α-SMA. Vascular smooth muscle cells in the vessel wall are stained by the α-SMA antibodies (brown). **E** Quantification of microvessels in vein grafts of ApoE^−/−^PFKFB3^fl/fl^ and ApoE^−/−^PFKFB3^ECKO^ mice. *P = 0.035 versus ApoE^−/−^PFKFB3^fl/fl^. Independent samples *t* test; *n* = 9 (ApoE^−/−^PFKFB3^fl/fl^) or *n* = 11 (ApoE^−/−^PFKFB3^ECKO^). **F** Quantification of α-SMA coverage of microvessels in vein grafts of ApoE^−/−^PFKFB3^fl/fl^ and ApoE^−/−^PFKFB3^ECKO^ mice. Independent samples *t* test did not show statistical significance (*n* = 8 for both groups)

**Fig. 5:**
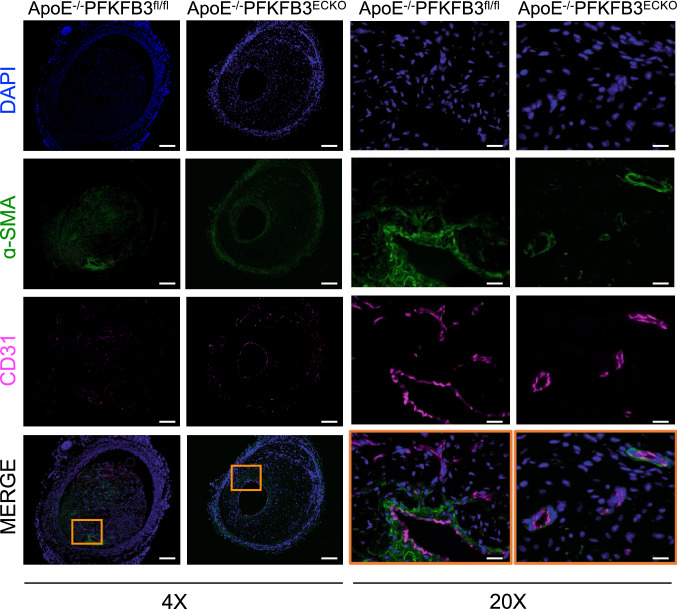
Examples of atherosclerotic lesions in vein grafts of ApoE^−/−^PFKFB3^fl/fl^ and ApoE^−/−^PFKFB3^ECKO^ mice stained with anti-CD31, α-SMA, and DAPI. An overlay of the three stainings is also shown as well as a magnification (20x) of the boxed areas showing mature and immature vessels. Scale bar = 500 μm (4x) or 100 μm (20x)

**Fig. 6:**
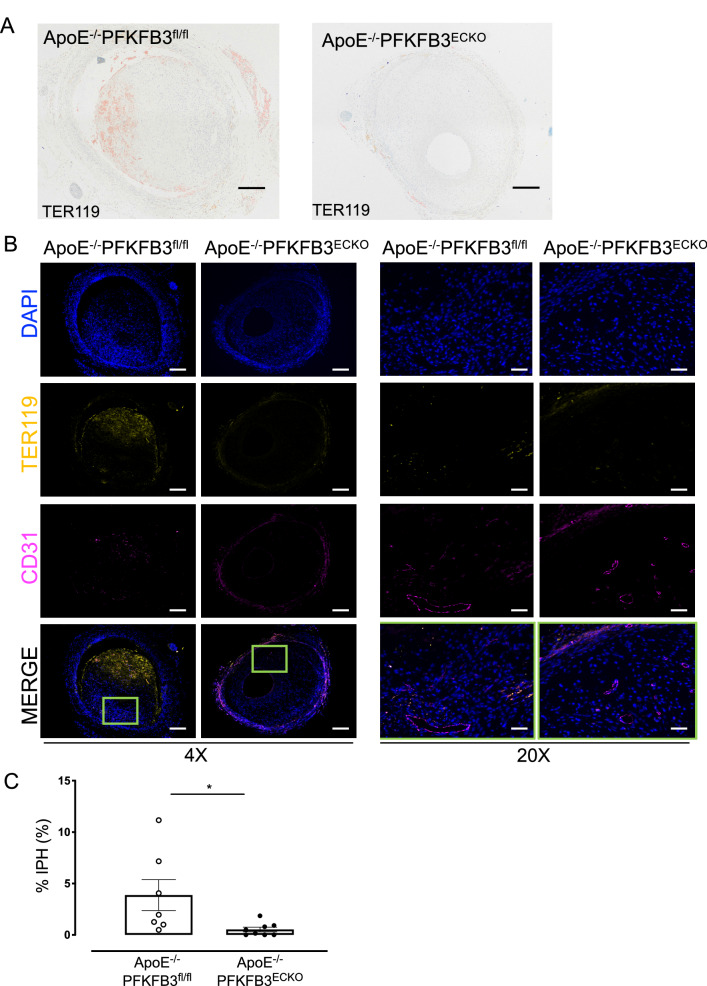
PFKFB3 deficiency in endothelial cells reduces intraplaque hemorrhages (IPH) in vein graft lesions. **A** Representative vein graft lesions stained with anti-TER-119. TER-119 staining (red) allows detection of erythrocytes. Scale bar = 200 μm. **B** Examples of atherosclerotic lesions in vein grafts of ApoE^−/−^PFKFB3^fl/fl^ and ApoE^−/−^PFKFB3^ECKO^ mice stained with anti-CD31, anti-TER-119, and DAPI. An overlay of the three stainings is also shown as well as a magnification (20x) of the boxed areas showing erythrocyte extravasation. Scale bar = 500 μm (4x) or 100 μm (20x). **C** Quantification of IPH. *P = 0.034 versus ApoE^−/−^PFKFB3^fl/fl^. Independent samples *t* test; *n* = 7 (ApoE^−/−^PFKFB3^fl/fl^) or *n* = 8 (ApoE^−/−^PFKFB3^ECKO^)

As shown by a CD31 staining, the number of microvessels per lesion was reduced by 62% in ApoE^−/−^PFKFB3^ECKO^ versus ApoE^−/−^PFKFB3^fl/fl^ mice (Fig. 4A, B, E). IP microvessels were further characterized with α-SMA staining to detect the presence of a VSMC layer around the microvessels (Fig. 4C, D). VSMC coverage was observed in vein grafts from both ApoE^−/−^PFKFB3^ECKO^ and ApoE^−/−^PFKFB3^fl/fl^ mice (Fig. 4F). There was no statistical difference between the two groups (*P* = 0.05), although a trend of higher VSMC coverage in ApoE^−/−^PFKFB3^ECKO^ mice was observed. The organization of the microvessel network was further assessed by immunofluorescence confocal microscopy (Fig. 5). Staining of graft lesions with CD31 antibody revealed IP microvessels that were often covered by VSMCs as demonstrated by α-SMA staining (Fig. 5). These findings suggest that IP microvessels are able to reach a significant level of structural and multicellular complexity. Anti-TER-119 staining showed more erythrocyte infiltration into the lesions of ApoE^−/−^PFKFB3^fl/fl^ mice as compared to lesions of ApoE^−/−^PFKFB3^ECKO^ mice (Fig. 6A–C), suggesting increased IP vessel leakage in ApoE^−/−^PFKFB3^fl/fl^ mice.

### PFKFB3 deficiency in ECs reduces lesion size of vein grafts

**Fig. 7:**
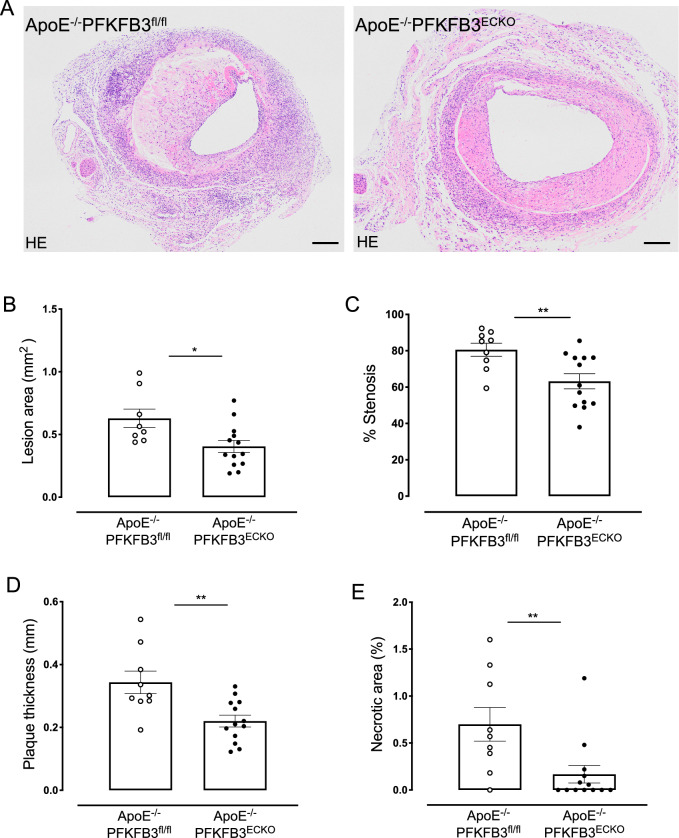
PFKFB3 deficiency in endothelial cells reduces the area, percentage stenosis, thickness, and necrotic area of vein graft lesions. **A** Representative cross sections of hematoxylin & eosin-stained vein grafts from ApoE^−/−^PFKFB3^fl/fl^ and ApoE^−/−^PFKFB3^ECKO^ mice. Scale bar = 200 μm. **B–E** Quantification of lesion area, percentage stenosis, thickness, and necrotic area of vein graft lesions. *P = 0.01, **P = 0.0095 versus ApoE^−/−^PFKFB3^fl/fl^. Independent samples *t* test; *n* = 8 (ApoE^−/−^PFKFB3^fl/fl^, panel B), *n* = 9 (ApoE^−/−^PFKFB3^fl/fl^, panel C–E), or *n* = 13 (ApoE^−/−^PFKFB3^ECKO^)

Twenty-eight days after vein graft surgery, the lesion area in the graft was decreased by 36% in ApoE^−/−^PFKFB3^ECKO^ mice as compared to ApoE^−/−^PFKFB3^fl/fl^ mice (Fig. 7A, B), suggesting a significant role of endothelial PFKFB3 in lesion formation and/or progression. Moreover, vein graft stenosis, lesion thickness, and necrotic area were significantly reduced in vein grafts of ApoE^−/−^PFKFB3^ECKO^ mice (Fig. 7C–E).

### PFKFB3 deficiency in ECs decreases macrophage infiltration

**Fig. 8:**
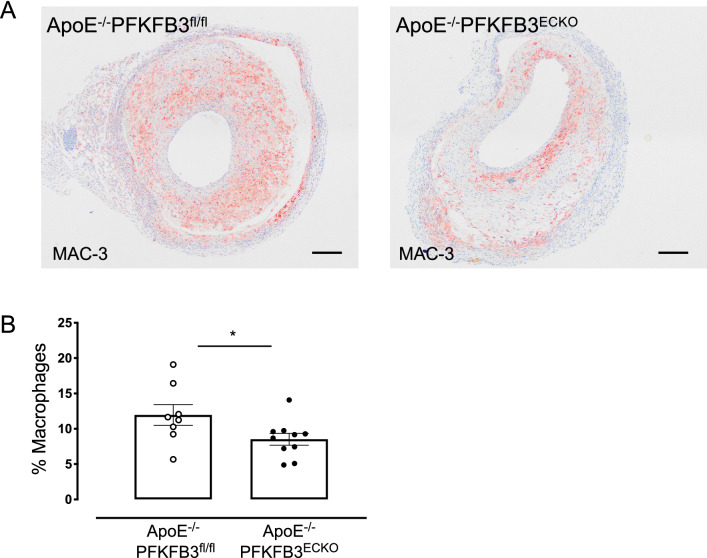
PFKFB3 deficiency in endothelial cells reduces macrophage infiltration in vein graft lesions. **A** Immunohistochemical detection of macrophages in representative vein graft lesions of ApoE^−/−^PFKFB3^fl/fl^ and ApoE^−/−^PFKFB3^ECKO^ mice using MAC-3 antibody. Scale bar = 200 μm. **B** Quantification of macrophages in vein graft lesions of ApoE^−/−^PFKFB3^fl/fl^ and ApoE^−/−^PFKFB3^ECKO^ mice. *P = 0.047 versus ApoE^−/−^PFKFB3^fl/fl^ mice. Independent samples *t* test; *n* = 8 (ApoE^−/−^PFKFB3^fl/fl^) or *n* = 10 (ApoE^−/−^PFKFB3^ECKO^)

Macrophage accumulation was mainly observed underneath the luminal EC or around the endothelium of microvessels in vein graft lesions of ApoE^−/−^PFKFB3^ECKO^ mice. In vein graft lesions of ApoE^−/−^PFKFB3^fl/fl^ mice, macrophages appeared much more diffuse in the vascular wall (Fig. 8A). Quantification of the macrophage infiltration showed a significant decrease in ApoE^−/−^PFKFB3^ECKO^ mice (Fig. 8B). Analysis of vascular cell adhesion molecule-1 (VCAM-1) expression at the luminal side of the vein graft did not reveal significant differences in ApoE^−/−^PFKFB3^ECKO^ mice as compared to ApoE^−/−^PFKFB3^fl/fl^ mice (Supplemental Figure S3 A–C).

### RT-PCR reveals an elevated M2 macrophage signature in vein grafts of ApoE^−/−^PFKFB3^ECKO^ mice

**Fig. 9:**
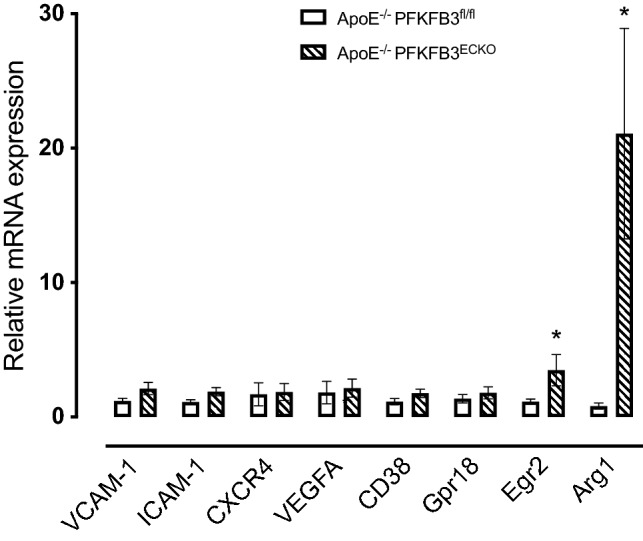
Real-time RT-PCR analysis of endothelial adhesion molecules, hypoxia markers, and M1/M2 macrophage markers in vein grafts of ApoE^−/−^PFKFB3^fl/fl^ and ApoE^−/−^PFKFB3^ECKO^ mice. Total RNA was isolated from vein grafts of ApoE^−/−^PFKFB3^fl/fl^ (PFKFB3^fl/fl^) and ApoE^−/−^PFKFB3^ECKO^ (PFKFB3^ECKO^) mice. Subsequently, mRNA expression of the endothelial adhesion molecules VCAM-1 (vascular cell adhesion molecule-1) and ICAM-1 (intercellular adhesion molecule-1), hypoxia markers CXCR4 (C-X-C chemokine receptor 4) and VEGFA (vascular endothelial growth factor A), M1 exclusive genes CD38 and G-protein coupled receptor 18 (Gpr18) as well as M2-specific markers Egr2 (Early growth response protein 2) and Arg1 (Arginase-1) was analyzed by real-time RT-PCR. *P < 0.05 versus PFKFB3^fl/fl^. Independent samples *t* test; *n* = 9 (ApoE^−/−^PFKFB3^fl/fl^) or *n* = 12 (ApoE^−/−^PFKFB3^ECKO^)

To obtain more insight into the mechanisms underlying the observed phenotype in vein grafts of ApoE^−/−^PFKFB3^ECKO^ mice, real-time RT-PCR reactions were performed (Fig. 9). Neither the expression of VCAM-1 and intercellular adhesion molecule-1 (ICAM-1) nor the expression of hypoxia markers C-X-C chemokine receptor 4 (CXCR4) and vascular endothelial growth factor A (VEGFA) significantly changed in vein grafts of ApoE^−/−^PFKFB3^ECKO^ mice as compared to vein grafts of ApoE^−/−^PFKFB3^fl/fl^ mice. mRNA levels of M1 macrophage markers CD38 and G-protein coupled receptor 18 (GPR18) did not change either. However, mRNA expression of the M2 markers Early growth response protein 2 (Egr2) and Arginase-1 (Arg1) were elevated in vein grafts of ApoE^−/−^PFKFB3^ECKO^ mice.

## Discussion

IP angiogenesis is frequently observed inside human vein graft lesions and is recognized as a contributing factor of plaque vulnerability [3, 6, 10, 11, 24]. In the present study, we crossed EC-specific PFKFB3 knockout mice with ApoE^−/−^ mice to investigate the role of EC glycolysis modulation in vein graft IP angiogenesis. To our knowledge, this is the first study using a conditional EC-specific PFKFB3 knockout mouse in the context of advanced atherosclerosis.

First of all, we did not observe any adverse effects or changes in general metabolism after PFKFB3 deletion in ECs. Circulating liver enzymes, blood glucose, insulin, and total cholesterol were not affected. Also glucose and insulin tolerance tests were similar in ApoE^−/−^PFKFB3^fl/fl^ versus ApoE^−/−^PFKFB3^ECKO^ mice. Ketone-body β-hydroxybutyrate was not changed in both groups. These findings suggest that PFKFB3 deletion in ECs does not lead to severe side effects or to a major metabolic switch in ApoE^−/−^ mice. In line with these findings, recent evidence indicates that the immune cell distribution in peripheral blood and lymphoid organs is unaffected after systemic treatment of mice with PFKFB3 inhibitor PFK158 [25].

Next, and in line with previous studies, we found that PFKFB3 deletion impaired vessel sprouting from aortic rings. Along these lines, ApoE^−/−^PFKFB3^ECKO^ mice showed a significantly reduced number of microvessels in vein graft lesions, albeit without a clear impact on hypoxia markers such as CXCR4 and VEGFA. Moreover, IP microvessels in ApoE^−/−^PFKFB3^ECKO^ showed a trend toward higher VSMC coverage and less leakage of erythrocytes inside the graft lesion. These findings are consistent with recent in vitro and in vivo observations showing that PFKFB3 inhibition reduces VE-cadherin endocytosis and promotes normalization of the endothelial barrier by tightening EC junctions [16]. Apart from PFKFB3 inhibition, we recently reported that also atorvastatin promotes IP vessel maturation in vein grafts by preventing VE-cadherin internalization and increasing pericyte coverage [9].

We also observed a reduction in plaque size in vein grafts of ApoE^−/−^PFKFB3^ECKO^ mice, which suggests that PFKFB3 may play a direct role in plaque progression. Such compelling possibility corresponds with data from in vitro studies showing that PFKFB3 is linked to pro-inflammatory signaling of ECs in response to blood flow shear stress. Indeed, turbulent blood flow in atheroprone regions leads to inhibition of Krüppel-like Factor 2 activity, which correlates with PFKFB3 upregulation, increased EC glycolysis, and inflammatory activation [26]. The importance of PFKFB3 in plaque progression has also been suggested by a recent study showing a positive correlation between PFKFB3 expression and an unstable plaque phenotype in both carotid and coronary plaques in humans [25]. Furthermore, administration of the PFKFB3 inhibitor PFK158 in mice led to a reduction in advanced plaques with a vulnerable phenotype and an increase in plaque stability [25]. The reduction of IP angiogenesis in ApoE^−/−^PFKFB3^ECKO^ mice, as described in this study, is also in line with previous findings in our group showing decreased IP angiogenesis following administration of the glycolysis inhibitor 3PO in a mouse model of advanced atherosclerosis [19]. In this perspective, it is worth mentioning that 3PO significantly reduced initiation of plaque formation in a preventive study design.

Most interestingly, we detected a reduction in the percentage of macrophage infiltration in vein graft lesions of ApoE^−/−^PFKFB3^ECKO^ mice. This finding is in agreement with the presence of crosstalk between EC metabolism and macrophages in pathological conditions, as previously reported [27, 28]. For example, in tumor settings a metabolic competition for glucose between EC and macrophages reduces EC hyperactivation and prevents abnormal vessel leakage [29]. However, both immunohistochemical stains and RT-PCR analysis of endothelial adhesion molecules did not reveal significant differences in ApoE^−/−^PFKFB3^ECKO^ mice as compared to ApoE^−/−^PFKFB3^fl/fl^ mice. PFKFB3 inhibition also abolishes the inflammatory response caused by lipoprotein(a) with concomitant attenuation of transendothelial monocyte migration in atherosclerotic plaques [30]. It is therefore possible that the observed reduction in macrophage infiltration *in vivo* is in part due to an improved restoration of EC junctions after PFKFB3 deletion, as mentioned above. Interestingly, vein grafts of ApoE^−/−^PFKFB3^ECKO^ mice revealed an elevated M2 macrophage signature. In particular the canonical M2 macrophage marker Arg-1 was strongly upregulated. Changes in CD38 and Gpr18, which are exclusive M1 markers [31] could not be demonstrated. M2 macrophages resolve inflammation and are usually associated with lesion regression, which corresponds to the improved phenotype of ApoE^−/−^PFKFB3^ECKO^ vein grafts. However, it is presently unclear what drives M2 polarization in our experimental settings. We previously reported that glycolysis inhibitor 3PO promotes a macrophage M2 phenotype by stimulating the expression of Arg1 and the exclusive M2 marker Egr2 [19]. Given that VE-cadherin-Cre (used to delete PFKFB3 in ECs) may also show recombinase activity in hematopoietic cells [32] we cannot rule out the possibility that also macrophages are (partially) PFKFB3 deficient. Importantly, myeloid knockdown of PFKFB3 does not affect the size and composition of plaques in atherosclerotic mice [33], suggesting that endothelial PFKFB3 deficiency and not myeloid PFKFB3 deficiency remains a key condition to suppress lesion formation and to obtain an improved lesion phenotype.

Altogether, our findings indicate that endothelial PFKFB3 plays a critical role in IP angiogenesis and lesion progression, and that PFKFB3 inhibition is a promising approach to prevent plaque development and to reduce the complications of vein bypass grafting.

## Supplementary Information

Below is the link to the electronic supplementary material.

Supplementary material 1 (PDF 9509.6 kb)

## Data Availability

The authors declare that all supporting data are available within the article (and its data supplement).
