# Supplementary material for: PFKFB3 gene deletion in endothelial cells inhibits intraplaque angiogenesis and lesion formation in a murine model of venous bypass grafting
Source: Angiogenesis. 2021 Aug 25;25(1):129–43. doi: 10.1007/s10456-021-09816-3 (PMC8813728; doi:10.1007/s10456-021-09816-3)
Supplement: Supplementary file 1 — Supplementary material 1 (PDF 9509.6 kb) [file 10456_2021_9816_MOESM1_ESM.pdf]

Supplemental material

PFKFB3 gene deletion in endothelial cells inhibits intraplaque angiogenesis and lesion formation in a murine model of venous bypass grafting

Paola Perrotta[1], Margreet R. de Vries[2], Bart Peeters[3], Pieter-Jan Guns[1], Guido R.Y. De Meyer[1], Paul H.A. Quax[2] and Wim Martinet[1]

[1] Laboratory of Physiopharmacology, University of Antwerp, Belgium
[2] Department of Surgery, Leiden University Medical Center, Leiden, the Netherlands
[3] Antwerp University Hospital, Laboratory Medicine, Belgium

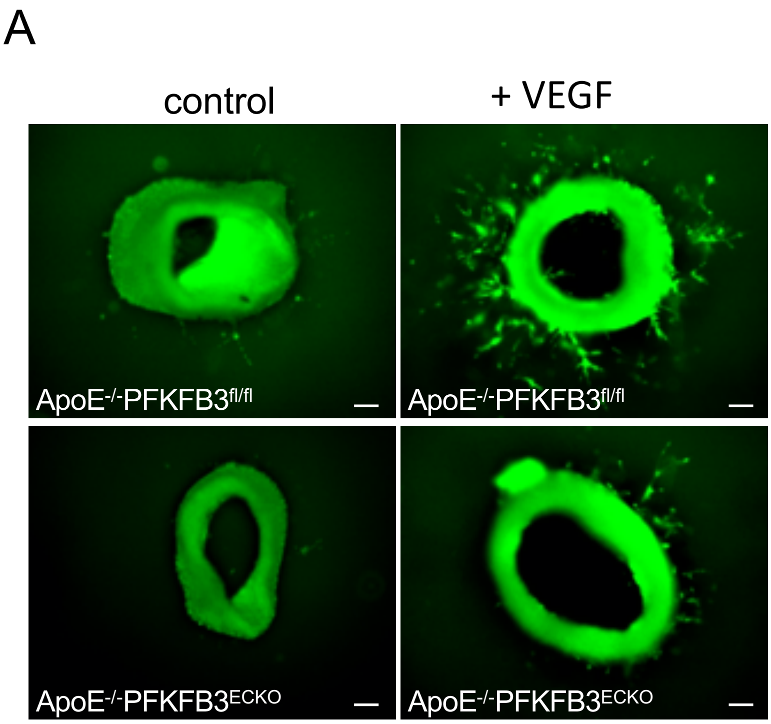

Figure S1. PFKFB3 deficiency in endothelial cells inhibits aortic sprouting. (A) Aortic rings from ApoE$^{-/-}$PFKFB3$^{fl/fl}$ and ApoE$^{-/-}$PFKFB3^{ECKO} mice were embedded in collagen type I and treated with Opti-MEM supplemented with 2.5% FBS (control) in the presence or absence of vascular endothelial growth factor (VEGF, 50 ng/mL). At day 6, rings were fixed and stained to delineate endothelial cells. Images of ring sprouting were obtained and quantified. Scale bar = 500 μm. (B) Sprouts were quantified, n=4 mice. ***P<0.0001 Two-way ANOVA, effect of PFKFB3^{ECKO}: **P<0.005, effect of VEGF: ***P<0.0001.

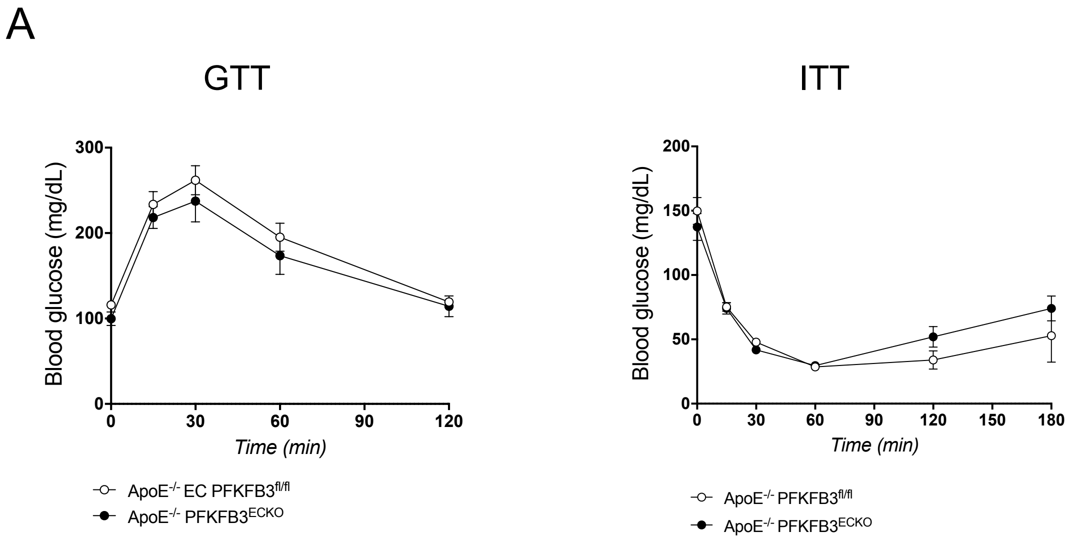

Figure S2. Glucose clearance in ApoE$^{-/-}$PFKFB3$^{fl/fl}$ and ApoE$^{-/-}$PFKFB3^{ECKO} mice after intraperitoneal injection with glucose or insulin. A glucose tolerance test (GTT) and insulin tolerance test (ITT) was performed after 12 weeks of western diet. Blood glucose was measured at different time points after injection of glucose (1 g/kg, i.p.) or insulin (1 U/kg, i.p.).

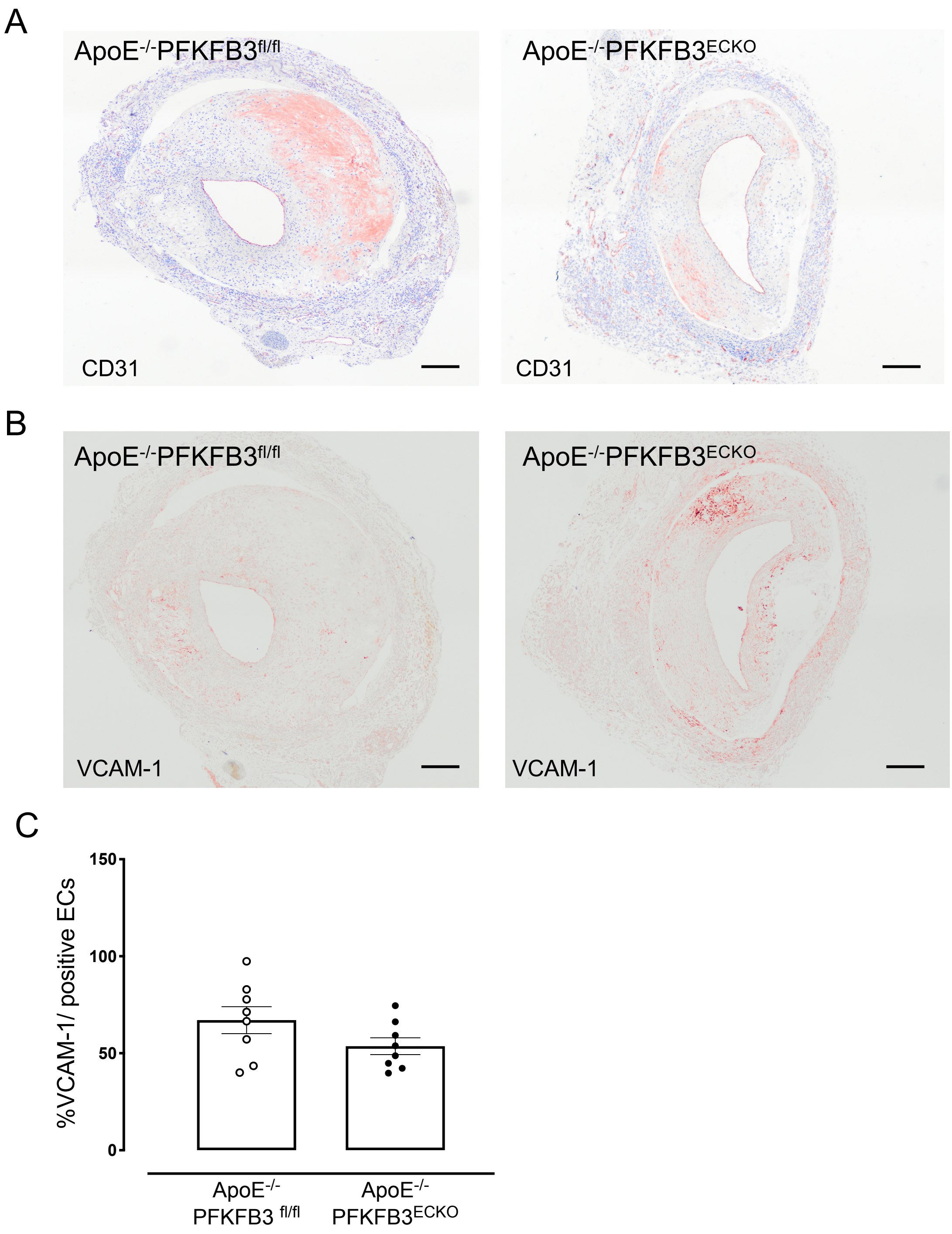

Figure S3. PFKFB3 deficiency in endothelial cells does not influence vascular cell adhesion molecule-1 (VCAM-1) expression in vein graft lesions. (A-B) Immunohistochemical detection of endothelial cells in representative vein graft lesions of ApoE$^{-/-}$PFKFB3$^{fl/fl}$ and ApoE$^{-/-}$PFKFB3^{ECKO} mice using CD31 and VCAM-1 antibody. Scale bar = 200 μm. (C) Quantification of VCAM-1 positive endothelial cells in vein graft lesions of ApoE$^{-/-}$PFKFB3$^{fl/fl}$ and ApoE$^{-/-}$PFKFB3^{ECKO} mice (Independent samples t test, n=8).
